# Antibiotics Use and Its Knowledge in the Community: A Mobile Phone Survey during the COVID-19 Pandemic in Bangladesh

**DOI:** 10.3390/antibiotics10091052

**Published:** 2021-08-29

**Authors:** Zubair Akhtar, Syeda Mah-E-Muneer, Md. Mahbubur Rashid, Md. Shakil Ahmed, Md. Ariful Islam, Sukanta Chowdhury, Zobaid Khan, Md. Zakiul Hassan, Khaleda Islam, Shahana Parveen, Nitish Debnath, Mahmudur Rahman, Fahmida Chowdhury

**Affiliations:** 1 Infectious Diseases Division, International Centre for Diarrhoeal Disease Research, Bangladesh (icddr,b), Dhaka 1212, Bangladesh; mahe@icddrb.org (S.M.-E.-M.); mahbubur.rashid@icddrb.org (M.M.R.); shakil.statru@gmail.com (M.S.A.); arif@icddrb.org (M.A.I.); sukanta@icddrb.org (S.C.); zhassan@icddrb.org (M.Z.H.); shahana@icddrb.org (S.P.); mrahman@globalhealthdev.org (M.R.); fahmida_chow@icddrb.org (F.C.); 2 Fleming Fund Country Grant to Bangladesh, DAI Global, LLC, House 3, First Floor, Road 23B, Gulshan 1, Dhaka 1212, Bangladesh; Zobaid_Khan@dai.com (Z.K.); Khaleda_Islam@dai.com (K.I.); Nitish_Debnath@dai.com (N.D.); 3 Nuffield Department of Medicine, University of Oxford, Oxford OX1 2JD, UK; 4 Global Health Development, EMPHNET, 69 Mohakhali, Dhaka 1212, Bangladesh

**Keywords:** antibiotic resistance, COVID-19, pandemic, antibiotic awareness, antibiotic use

## Abstract

The general population has been excessively using antibiotics during the COVID-19 pandemic. Therefore, the use of antibiotics for any reported illnesses in the preceding four weeks and knowledge of antibiotics among the general population in the community were assessed for possible interventions. A mobile phone survey among a general population across eight administrative divisions of Bangladesh was conducted during January–March 2021. Reported illness episodes irrespective of COVID-19 in the preceding four weeks of the interview, use of antibiotics for the illnesses, and knowledge on antibiotics among the general population were recorded. Descriptive analyses were performed. We randomly interviewed 1854 participants, with a mean age of 28.5 years (range: 18–75 years); 60.6% were male. Among all participants, 86.3% (95% CI: 84.7–87.8) heard names of antibiotics, but only 12.1% reported unspecified harmful effects, and 3.5% reported antimicrobial resistance when antibiotics were taken without a physician’s prescription. Among 257 (13.9%) participants, who consumed medicines for their recent illness episode, 32.7% (95% CI: 27.2–38.6) reported using antibiotics. Of those who could recall the names of antibiotics prescribed (n = 36), the most frequently used was azithromycin (22.2%) followed by cefixime (11.1%) and ciprofloxacin (5.6%). Our findings show an increased antibiotic use for illnesses reported in the preceding four weeks and an elevated knowledge at the community level during the COVID-19 pandemic compared with the pre-pandemic period.

## 1. Introduction 

Modern healthcare is predominantly reliant on antibiotic treatment [1], but there has been a phenomenal and imprudent use of antibiotics leading to the advent of resistant strains of bacteria [2,3]. To address the antimicrobial resistance (AMR) as a whole, with more emphasis on antibiotic resistance, the World Health Organization (WHO) initiated a range of AMR-related activities, including the development of the Global Action Plan on Antimicrobial Resistance (GAP-AMR) by the 68th World Health Assembly in May 2015 [4]. Published literatures exhibit a high proportion of inappropriate use of antimicrobials, and, as a result, optimizing the use of antimicrobial agents is one of the five key strategic objectives outlined in the GAP-AMR [4]. 

In late 2019, a novel coronavirus (SARS-CoV-2) was identified as the cause of an outbreak of disease named COVID-19 (coronavirus disease 2019), causing pneumonia in severe cases [5] further complicating as an acute respiratory distress syndrome (ARDS), a hyperinflammatory state and ultimately a multiorgan dysfunction with fatal consequences [6,7]. It was subsequently characterized as a pandemic on 11 March 2020 by the WHO [8]. Despite being a viral disease, it mimicked clinical symptoms of bacterial pneumonia; hence, different antimicrobials, especially antibiotics, were used as empirical therapy [9,10,11,12,13]. Furthermore, during the initial stages of the pandemic, there was a lack of proper antivirals with proven efficacy, and this, compounded by the anxiety and uncertainty of available treatment, led to the empirical but rather widespread and excessive use of antibiotics [13]. A study in Italy showed that, during lockdown for COVID-19, there was a relevant reduction in antibiotic consumption among children, due to closed daycare centers and schools, except for a relative increase in azithromycin use in adults [14]. However, there was no strong justification for routine use of azithromycin for reducing time to recovery or risk of hospitalization from the suspected COVID-19 in the community [15]. 

During the COVID-19 pandemic, the general population has likely been sensitized about using antibiotics for treating COVID-19. According to web-based surveillance on the COVID-19 pandemic and antibiotics used in Malaysia, 37% of the participants were aware that using antibiotics could not speed up recovery from all infections [16]. Still, 49% of the respondents reported that antibiotics were effective against bacterial infection only [16]. Dispensing of antibiotics was increased profoundly in Egypt during the early period of the COVID-19 pandemic without proper clinical evaluation, and azithromycin, ceftriaxone, and linezolid were the major antibiotics used [17]. Approximately, 93% of the presumptive COVID-19 patients received antibiotics with official prescriptions and without prescriptions, of which 18% comprised of pharmacist and patient’s recommendations [17]. Before the COVID-19 pandemic, studies in Bangladesh reported that 48% of adult respondents heard about antibiotics, 70% of children of those parents who were aware of antibiotics had received it previously, and 28% of study participants took antibiotics before presenting at the hospital for acute febrile illness [18,19]. During the first wave of the COVID-19 pandemic in Bangladesh, a study among suspected COVID-19 patients found that the use of antibiotics was 92% during their overall suspected phase. Among them, 89% were prescribed antibiotics on hospital admission, while 47% of COVID-19 suspected patients received antibiotics 24 h before hospital admission [20]. A prime contributor to antibiotics overuse in Bangladesh is perhaps the availability of over-the-counter dispensed antibiotics through unregulated drug stores (pharmacies) [19,21]. Therefore, assessing the use of antibiotics and the relevant knowledge on antibiotics among the general population irrespective of hospitalization, especially during the COVID-19 pandemic, is imperative in order to formulate policy interventions regarding the rational use of antibiotics. 

Higher-income countries have used telephone surveys to collect real-time data on population-level estimates of health and demographics [22,23,24]. Bangladesh had a mobile phone teledensity of 103% in 2021 [25], with over 175 million subscribers registered in May 2021 [26]. We utilized this opportunity and conducted a mobile-phone-based survey to obtain real-time data during the COVID-19 pandemic. In this study, we aimed to assess antibiotic use for any reported illnesses in the preceding four weeks and knowledge regarding antibiotics among the general population in Bangladesh.

## 2. Methods

### 2.1. Study Design and Study Population

We conducted a cross-sectional mobile-phone-based survey during the COVID-19 pandemic for eight weeks between mid-January 2021 and mid-March 2021 to assess antibiotic use and relevant knowledge on antibiotic use in Bangladesh utilizing the Computer-Assisted Telephone Interviewing (CATI) system [27,28]. The target population was adult individuals (aged 18 years and above) with a mobile phone subscription across all eight administrative divisions in Bangladesh to ensure a national geographical representation. 

### 2.2. Data Collection

A mobile phone database (containing only numbers and locations from where the subscription was registered) of a mobile-phone survey platform of the Programme for Emerging Infections at International Centre for Diarrhoeal Disease Research, Bangladesh (icddr,b) was used as the sample population. The CATI software randomly picked up mobile phone numbers from that database for the interviews. Two interviewers made phone calls to random numbers from 9.00 AM to 5.00 PM throughout the weekdays (Sunday to Thursday). If any respondent could not speak during the call time but agreed to take part at a later convenient date and time, the interviewer fixed an appointment for the interview, especially on weekends. On weekends (Friday and Saturday), phone calls were made from 10:00 AM to 12:00 PM for those working adults who had earlier scheduled the interviews. Data through mobile phone calls were collected using a tablet device and entered in real-time into the icddr,b server. Each phone call started with greetings stating the objective of the call and informing that the names of the interviewee would not be asked, data would be confidential, and an age screening question would be asked followed by a verbal consent to start the interview. Interviews only progressed if the respondent agreed to participate and was 18 years of age or older. For social acceptance during the interview, we used the common term “corona” instead of “COVID-19”.

### 2.3. Data Collection Tool 

We used a structured questionnaire to register socio-demographic variables followed by the experience of general illnesses, including any COVID-19 disease in the preceding four weeks and the healthcare-seeking pattern during the pandemic period. Subsequently, the frequency of healthcare-seeking for the illness was documented, including the frequency of antibiotic use. Information on knowledge on antibiotics such as any antibiotic name recalled spontaneously, the name with the duration of antibiotic, if used, and any adverse effects known if taken without a doctor’s prescription were also collected. In total, 60 pilot interviews were conducted in two rounds (30 in each round) before initiating the study to validate the tool used, and study variables were restructured based on feedback from the pilot interviews. A response rate and a cooperation rate (defined later) of the survey conducted were determined. 

### 2.4. Computer-Assisted Telephone Interviewing (CATI) System

The CATI system integrated the data collection tool with the mobile phone database over the internet. It also provided an interface on a handheld tablet device for making calls, scheduling repeat calls, selecting mobile interviewee numbers randomly, and logging the interviewers’ activities [29]. In addition, the system had inbuilt algorithms to perform automated variables’ check and facilitated direct data entry. On a typical workday, the interviewer logged into the system with a unique ID and password on a tablet device into the web interface of the CATI system, and it randomly selected the phone numbers to be dialed. Once the call was connected, the interview progressed with the interviewer registering entries concurrently in the data server where all the responses were stored. Subsequently, more interviews were conducted in the same manner. 

### 2.5. Sample Size Calculation

According to recent studies, 48% of the respondents were found to hear about antibiotics in 2018, and 40% of the respondents used antibiotics for respiratory illness during 2012–2013 [18,30]. Based on the statistics above, we conservatively expected that antibiotics might be known and consumed by 20% general population consisting of those with illnesses, fear of having illnesses, and/or presumed illnesses in Bangladesh. Therefore, assuming a 95% confidence level with 5% absolute precision and 20% expected to be aware and used antibiotics, we required a minimum sample size of 246 individuals. This sample size would be sufficient to detect any prevalence of antibiotic awareness and use of 20% to 80% with the 95% confidence level [31]. Since we wanted to have a national representative sample, we opted to cover each of the eight administrative divisions of Bangladesh. Therefore, we targeted a minimum sample size of (246 × 8 = 1968) respondents across Bangladesh. 

### 2.6. Data Analysis

From the CATI software system, we calculated the response rate and cooperation rates as following:

$$\mathrm{Response}\;\mathrm{rate}\;(\%)\;=\;\frac{\mathrm{Calls}\;\mathrm{received}\;}{\mathrm{Calls}\;\mathrm{dialed}}\times 100$$

$$\mathrm{Cooperation}\;\mathrm{rate}\;(\%)\;=\;\frac{\mathrm{Interviews}\;\mathrm{completed}}{\mathrm{Calls}\;\mathrm{received}}\times 100$$

Interview data were stored in icddr,b local server. Stata v.13 (StataCorp LP, College Station, TX, USA) was used to perform descriptive analysis to summarize the categorical variable based on frequency distribution with percentage and 95% confidence interval. For continuous variables, we used descriptive statistics (mean, standard deviation (SD), and range) for symmetric distribution and median and interquartile range (IQR) for asymmetric distribution. We also used Python 3.6 to create maps illustrating study findings.

## 3. Results

**Figure 1:**
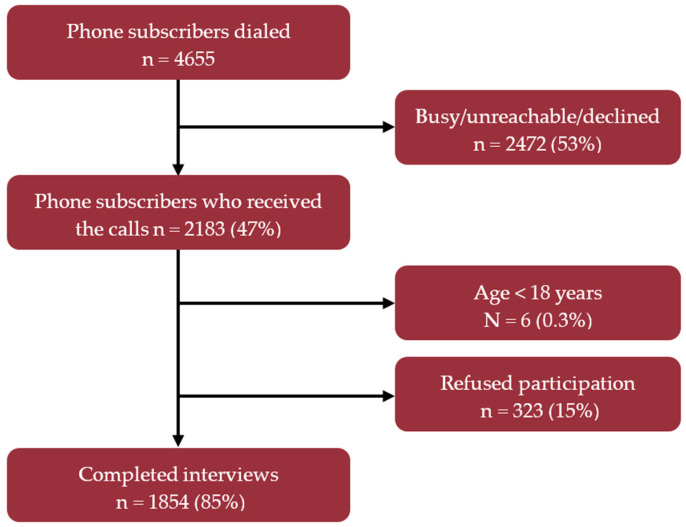
CONSORT diagram showing participants’ enrollment, response rate, and cooperation rate of mobile phone survey during January–March 2021 in Bangladesh.

A total of 2183 respondents were reached in our study. On average, there were 280 calls made per week and 1854 complete interviews conducted from 62 districts of Bangladesh. Therefore, the cooperation rate was 85% for this study (Figure 1). The average duration of calls was 8 (range: 3–16) min.

Among the 1854 complete interviews conducted, 60.6% were male, and the mean age was 28.5 years ranging from 18 to 75 years. The majority (45.4%) of them were students, followed by homemakers (23.3%) and service holders (16.7%). The median household size was 5 members, and 50.7% were living in urban areas. The division-wise response proportions are listed in Table 1.

Among those consuming medicines (n = 257, 13.9%) for their general illness reported in the preceding four weeks, 4.7% did not know whether they were taking antibiotics together, with another 4.7% missing/no responses. The proportion of respondents using antibiotics was 32.7% (95% CI: 27.2–38.6, n = 84) for general illnesses in the preceding four weeks. Among the COVID-19 patients (n = 16), this proportion of use of antibiotics was only 12.5% (n = 2). Purchase of antibiotics (n = 84) was advised by the seller at pharmacies (33.3%) as over-the-counter sales, on the prescription of formal private clinics (21.4%), on the prescription of formal (MBBS/MD) doctors (15.5%), and those from public healthcare facilities (14.3%). Thirteen percent of the respondents also reported purchasing antibiotics advised by village doctors (quacks). Among respondents purchasing antibiotics, 17.9% reported purchasing a partial number of antibiotics of the prescribed antibiotics. Of those who could recall the names of antibiotics (n = 36), the most frequently consumed was azithromycin (22.2%) followed by cefixime (11.1%) and ciprofloxacin (5.6%) (Table 2).

About one-fifth (18.5%, n = 343) of all the respondents reported symptoms of general illness in the past four weeks of the interview. Among those who reported illness, runny nose (9.3%) was mainly reported, followed by fever (2.3%), cough (1.6%), and diarrhea (1.2%). The COVID-19 specific symptom of anosmia/ageusia, sore throat, and headache were reported by 0.7%, 0.6%, and 0.6% of the respondents, respectively. Only 13.9% (n = 257) of all study participants sought healthcare for their illness. Among them, 46.7% went to medicine shops (pharmacies) for treatment. The healthcare-seeking respondents also sought healthcare from private clinics (16.0%), formal (MBBS/MD) doctors (12.1%), took self-medication (8.6%), went to village doctors (7.8%), followed by resorting to public healthcare facilities (7.0%) for their illnesses. The majority reported consuming one−three medicines (77.8%), four−five medicines (12.1%), and six medicines or more (2.3%) for their recent episode of illness in the last four weeks. Two percent of respondents reported hospitalizations due to any disease among those who sought healthcare (Table 3). 

**Figure 2:**
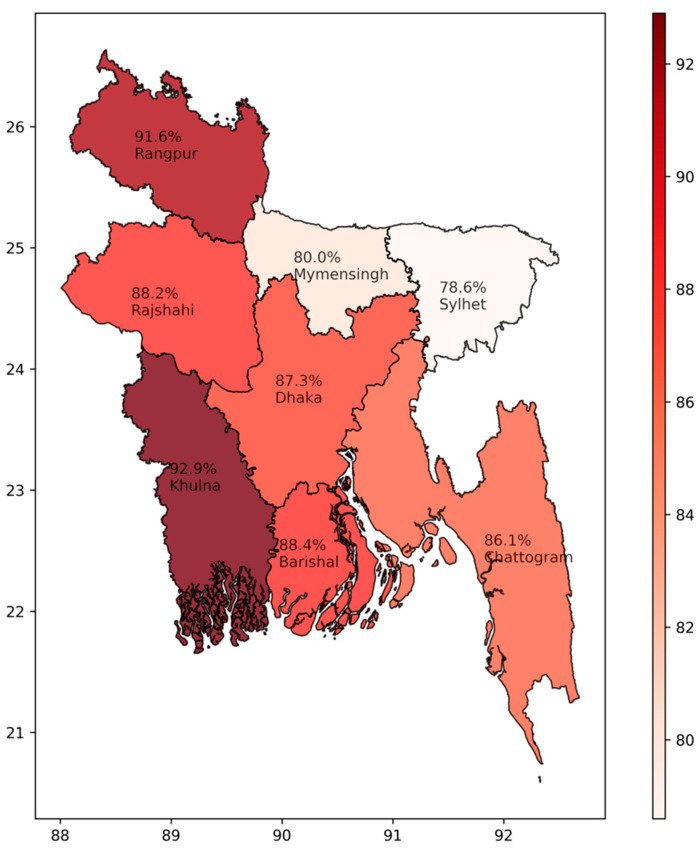
Proportion of the general population knowing about antibiotic during the COVID-19 pandemic in Bangladesh.

Most (86.3%, 95% CI: 84.7–87.8, n = 1600) of the respondents heard names of antibiotics in their lifetime, and the division-wise proportionate responses are illustrated in Figure 2. They could state names of azithromycin (12.0%), ciprofloxacin (6.6%), cefixime (4.8%), and amoxycillin (2.8%), spontaneously. When interviewed for dosage of antibiotics, 48.3% (n = 772) reported that they knew the dosage and reported antibiotic use duration as one week (68.9%), three to five days (17.2%), two weeks (9.1%), more than two weeks (3.4%), and less than three days (1.4%). Unspecified harmful effects were stated by 12.1% of the respondents, kidney problems by 5.5% of the respondents, and 3.5% of the respondents reported antibiotic resistance as the effect of taking antibiotics without consulting a physician (Table 4).

Among all the respondents, 6% (n = 111) reported that they underwent COVID-19 testing, and most (81.1%, 95% CI: 72.8–87.3) availed public facilities for it. Among those who tested for COVID-19 (n = 111), 14% (n = 16) were tested positive, and 4% (n = 4) were hospitalized. A small proportion (8%, n = 141) of all respondents stated that their family members underwent COVID-19 testing; 13% tested positive, while 9% did not respond to this question, and 5% of COVID-19 affected family members were admitted to the hospital. Five deaths (4%) among family members with COVID-19 disease were also reported (Table 5).

## 4. Discussion

Our study found 32.7% (95% CI: 27.2–38.6) of the general population who reported illness in the preceding four weeks of the interview using antibiotics in the community. This proportion of antibiotic use for illnesses was higher than a pre-pandemic study in a community setting in Bangladesh during 2018, reporting (21%) antibiotics use within the last month [18]. The COVID-19 pandemic may have influenced this rise in the proportion of antibiotic use in Bangladesh. However, the proportion of antibiotics used among COVID-19 patients was much low (12.5%) in our findings. However, determining the proportion of antibiotic use among COVID-19 cases was not the study’s objective. Yet, this study finding is much lower than 47% among the suspected COVID-19 cases reported in a previous hospital-based study from Bangladesh [20]. The proportion of antibiotics used for COVID-19 respondents in our study is also much lower than the study conducted in Egypt during the COVID-19 pandemic [17]. This may be likely to the fact that a very low number of COVID-19 cases were detected through this survey.

This study also measured the relevant knowledge regarding antibiotics irrespective of any respondents suffering from COVID-19 illness. The basic knowledge regarding antibiotics was very high among our survey respondents. Our study found a very high proportion (86.3%, 95% CI: 84.7–87.8) of respondents reported to know names of antibiotics compared with the previously reported proportion of 48% in Bangladesh before the COVID-19 pandemic [18]. Furthermore, azithromycin, a macrolide antibiotic, was found as the most frequently (12%) recalled/reported antibiotic. This is perhaps because azithromycin was the most commonly prescribed antibiotics by physician’s advice and self-medication during the COVID-19 pandemic situation in Bangladesh [32] and other countries in Europe and Asia [33]. An online survey conducted at the end of March 2020 on 6227 physicians in 30 countries revealed that, after some common analgesics, azithromycin was the second-highest prescribed drug for COVID-19 [34]. Earlier reports on antibiotics awareness in Bangladesh stated the most frequent (11%) antibiotic reported to be a fluoroquinolone named ciprofloxacin [35]. Although our study participants mentioned several unspecified harmful effects of antibiotics if taken without a physician’s prescription, very few (4%) could expressly state about antibiotic resistance. This rate is much lower than previous reports from Bangladesh, where 60% of the respondents stated antibiotic resistance [35]. Overall, our findings illustrate that the general population was more knowledgeable about antibiotics during the COVID-19 pandemic compared with the pre-pandemic period. 

Drug shops (pharmacies) contributed to the lion’s share (33.3%) of antibiotic dispensing without a prescription, and village doctors also contributed a handsome proportion (13.1%) in prescribing antibiotics in our study. Recent past studies in Bangladesh reported proportions as high as 43% [36] and 16% from drug shops (pharmacies), and 18% from village doctors [35]. Bangladesh’s common practice is to fetch over-the-counter medicines and even antibiotics regardless of consulting any qualified healthcare providers and the socio-economic status and education of the buyer [37,38]. Our findings and this intrinsic nature of the population regarding procuring antibiotics raise a flag to immediately adopt and strengthen “prescription-only from formal providers” for antibiotic purchase [20].

The antibiotic azithromycin was the most frequently prescribed/used, followed by cefixime—a third-generation cephalosporin. For treating suspected or confirmed mild-to-moderate COVID-19 cases without clinical suspicion of bacterial infections, antibiotics were discouraged from using for empirical treatment by WHO [39]. However, due to overburdened laboratories for testing microbiological samples and lack of recommended antiviral therapy for COVID-19 infection, there was an increased empirical use of antimicrobials, including broad-spectrum antibiotics, by the clinicians [13,40]. In Spain, a biphasic use of antibiotics was observed during the COVID-19 pandemic during 2020, along with amoxicillin/clavulanic acid and broad-spectrum antibiotics in public referral hospitals [40]. Physicians reported the widespread use of broad-spectrum antibiotics in 23 countries [9]. Moreover, the most common antibiotic classes prescribed were macrolides, fluoroquinolones, β-lactam/β-lactamase inhibitors, and cephalosporins in North America, Europe, China, and Asia [33]. The use of macrolide and cephalosporins were similar to the results from a recent study conducted across Bangladesh during the first wave of the COVID-19 pandemic, where macrolide was the most frequently (27%) used antibiotic followed by cephalosporin (16%) among suspected COVID-19 patients before hospital admission [20]. However, both azithromycin and cefixime belong to the Watch group antibiotic, which is not recommended according to the WHO AWaRe classification/tool [41]. This non-compliance in antibiotic use also flags a concern for antimicrobial resistance. 

Although we were able to cover respondents from across eight administrative divisions of Bangladesh, while interpreting our study findings, several limitations must be kept in consideration. The majority of our study respondents were males aged 18–40 years; therefore, our sampled respondents may not represent Bangladesh’s demographic profile. Furthermore, antibiotics were more likely to be used in extremes of age while we had no respondents <5 years, and only 4.2% were above 50 years. It was a survey based on mobile phone, and hence there may be recall bias in the responses leading to data loss and distortion. Furthermore, knowledge questions on antibiotics were limited to basic ones such as names, dosage, and harmful effects. Lastly, there were minimal opportunities to cross-validate responses, and only 5% of the complete interviews were randomly cross checked by repeat interviews. Overall, our results may be an underestimate of our actual prevalence of antibiotic use and knowledge.

In conclusion, our study findings underscored the increased proportion of antibiotic use for recent illnesses and raised knowledge about antibiotics at the community level during the COVID-19 pandemic in Bangladesh. Overuse of antibiotics, widespread availability, and generalized access to all types of antibiotics as over-the-counter drugs in the community surface raise concerns for antibiotic resistance in the near future. A robust monitoring system supported by policy and law is highly recommended to delimit over-the-counter antibiotic sales. Together with building community awareness on AMR, precisely due to the irrational use of antibiotics, it is imperative to promote, standardize, and strengthen antimicrobial stewardship within the health system of Bangladesh.

## Figures and Tables

**Table 1:** Demographic characteristics of the participants of mobile phone survey during the COVID-19 pandemic from January 2021 to March 2021 in Bangladesh.

Demographic Characteristics (N = 1854)	N	% (95% CI)
Age (Years)		
18–30	1239	66.8 (64.7, 68.9)
31–40	347	18.7 (17, 20.6)
41–50	191	10.3 (9, 11.8)
50+	77	4.2 (3.3, 5.2)
Mean age in years (Range)	28.5 (18–75)	
Gender		
Male	1124	60.6 (58.4, 62.8)
Female	649	35.0 (32.9, 37.2)
Not willing to disclose	81	4.4 (3.5, 5.4)
Occupation		
Student	842	45.4 (43.2, 47.7)
Homemaker	432	23.3 (21.4, 25.3)
Service holder	309	16.7 (15, 18.4)
Businessman	104	5.6 (4.7, 6.8)
Skilled worker	50	2.7 (2.1, 3.5)
Farmer	43	2.3 (1.7, 3.1)
Unemployed	34	1.8 (1.3, 2.6)
Retired	14	0.8 (0.5, 1.3)
Doctor	7	0.4 (0.2, 0.8)
Others	12	0.6 (0.4, 1.1)
No Response	7	0.4 (0.2, 0.8)
Median household size, members (IQR)	5 (4–6)	
Place of residence		
Urban	939	50.6 (48.4, 52.9)
Rural	876	47.2 (45, 49.5)
Not willing to disclose	39	2.1 (1.5, 2.9)
Division name of the residence		
Dhaka	535	28.9 (26.8, 31)
Chattogram	403	21.7 (19.9, 23.7)
Khulna	353	19.0 (17.3, 20.9)
Rangpur	202	10.9 (9.6, 12.4)
Barishal	199	10.7 (9.4, 12.2)
Sylhet	84	4.5 (3.7, 5.6)
Rajshahi	17	0.9 (0.6, 1.5)
Mymensingh	15	0.8 (0.5, 1.3)
Missing/No response	46	2.5 (1.9, 3.3)

**Table 2:** Use of antibiotics among the general population who reported illness in the preceding four weeks of the interview during the COVID-19 pandemic in Bangladesh.

Antibiotic Use	N	% (95% CI)
Antibiotics used for reported illness (n = 257)		
Yes	84	32.7 (27.2, 38.6)
No	149	58.0 (51.9, 63.9)
Don’t know	12	4.7 (2.7, 8)
Missing/no response	12	4.7 (2.7, 8)
Source of antibiotic prescription (n = 84)		
Pharmacy	28	33.3 (24.2, 43.9)
Formal private clinic	18	21.4 (14, 31.3)
Formal (MBBS/MD) doctor	13	15.5 (9.3, 24.7)
Public healthcare facility	12	14.3 (8.4, 23.3)
Quack, village doctor	11	13.1 (7.5, 21.9)
Self-medication	2	2.4 (0.7, 8.3)
Recalled names of antibiotics prescribed (n = 84)		
Yes	36	42.9 (32.8, 53.5)
No	15	17.9 (11.1, 27.4)
No response/don’t know	33	39.3 (29.5, 50)
Names of the prescribed antibiotics recalled (n = 36)		
Azithromycin	8	22.2 (11.7, 38.1)
Cefixime	4	11.1 (4.4, 25.3)
Ciprofloxacin	2	5.6 (1.5, 18.1)
Co-trimoxazole	1	2.8 (0.1, 14.2)
Flucloxacillin	1	2.8 (0.1, 14.2)
Metronidazole	1	2.8 (0.1, 14.2)
Tetracycline	1	2.8 (0.1, 14.2)
Purchase of prescribed antibiotics (n = 84)		
Full course purchased	69	82.1 (72.6, 88.9)
Partial purchase	15	17.9 (11.1, 27.4)
Duration of antibiotics taken (n = 84)		
3–5	37	44.0 (33.9, 54.7)
6–7	27	32.1 (23.1, 42.7)
8–14	6	7.1 (3.3, 14.7)
14+	6	7.1 (3.3, 14.7)
<3	5	6.0 (2.6, 13.2)
Missing/No response	3	3.6 (1.2, 10)
Median duration of antibiotics taken (IQR)	5 (3–7)	
Antibiotics used for COVID-19 positive (n = 16)		
Yes	2	12.5 (3.5, 36)
No	1	6.3 (0.3, 28.3)
Missing/No response	13	81.3 (57, 93.4)

**Table 3:** General illness among the general population during the COVID-19 pandemic in Bangladesh.

Symptoms Reported for the Current Episode of Illness (N = 1854)	N	% (95% CI)
Runny nose	173	9.3 (8.1, 10.7)
Fever	42	2.3 (1.7, 3)
Cough	29	1.6 (1.1, 2.2)
Loose motion/dysentery	23	1.2 (0.8, 1.9)
Loss of smell/taste	13	0.7 (0.4, 1.2)
Sore throat	11	0.6 (0.3, 1.1)
Headache	11	0.6 (0.3, 1.1)
Injury/accident	8	0.4 (0.2, 0.8)
Urinary tract infection	7	0.4 (0.2, 0.8)
Allergy	5	0.3 (0.1, 0.6)
Difficulty breathing	4	0.2 (0.1, 0.6)
Any others	17	0.9 (0.6, 1.5)
No symptoms	1454	78.4 (76.5, 80.2)
Missing/no response	57	3.1 (2.4, 4)
Healthcare seeking behavior for the current episode of illness (n = 1854)		
Healthcare sought		
Yes	242	13.1 (11.6, 14.7)
Self-medication	15	0.8 (0.5, 1.3)
No	1546	83.4 (81.6, 85)
Missing/no response	51	2.8 (2.1, 3.6)
Healthcare seeking point (n = 257) *		
Pharmacy	120	46.7 (40.7, 52.8)
Formal private clinic	41	16.0 (12, 20.9)
Formal (MBBS/MD) doctor	31	12.1 (8.6, 16.6)
Self	22	8.6 (5.7, 12.6)
Quack, village doctor	20	7.8 (5.1, 11.7)
Public healthcare facility	18	7.0 (4.5, 10.8)
Followed the previous prescription	0	-
Others (NGO and Homeopathy)	5	1.9 (0.8, 4.5)
Hospitalization required (n = 257)		
Yes	5	1.9 (0.8, 4.5)
No	252	98.1 (95.5, 99.2)
Medicine taken (n = 257)		
Yes	246	95.7 (92.5, 97.6)
No	11	4.3 (2.4, 7.5)
Number of medicines taken (including Antibiotic) (n = 257)		
1–3	200	77.8 (72.4, 82.5)
4–5	31	12.1 (8.6, 16.6)
>6	6	2.3 (1.1, 5)
Missing/no response	20	7.8 (5.1, 11.7)

* inclusive of those (n = 257) who sought health care (n = 242) and self-medicated (n = 15).

**Table 4:** Knowledge about antibiotics among the general population during the COVID-19 pandemic in Bangladesh.

Antibiotic Knowledge	N	% (95% CI)
Ever heard any name of antibiotics (N = 1854)		
Yes	1600	86.3 (84.7, 87.8)
No	254	13.7 (12.2, 15.3)
Name of antibiotics recalled spontaneously (n = 1600)		
Azithromycin	192	12.0 (10.5, 13.7)
Ciprofloxacin	106	6.6 (5.5, 8)
Cefixime	76	4.8 (3.8, 5.9)
Amoxicillin	45	2.8 (2.1, 3.7)
Flucloxacillin	20	1.3 (0.8, 1.9)
Cefradine	9	0.6 (0.3, 1.1)
Metronidazole	4	0.3 (0.1, 0.6)
Ceftriaxone	3	0.2 (0.1, 0.5)
Tetracycline	2	0.1 (0, 0.5)
None	1143	71.4 (69.2, 73.6)
Knowledge of antibiotics use duration (n = 1600)		
Yes	772	48.3 (45.8, 50.7)
No	821	51.3 (48.9, 53.8)
Missing/no response	7	0.4 (0.2, 0.9)
Knowledge of duration (days) of antibiotics use (n = 772)		
1–7	532	68.9 (65.6, 72.1)
3–5	133	17.2 (14.7, 20.1)
8–14	70	9.1 (7.2, 11.3)
14+	26	3.4 (2.3, 4.9)
<3	11	1.4 (0.8, 2.5)
Median (IQR)	7 (3–7)	
Knowledge of side effects of taking antibiotics without consulting a doctor (n = 1600)		
Harmful effects (unspecified)	194	12.1 (10.6, 13.8)
Kidney problem	88	5.5 (4.5, 6.7)
Antibiotic resistance	56	3.5 (2.7, 4.5)
Liver problem	29	1.8 (1.3, 2.6)
Heart problem	21	1.3 (0.9, 2)
Headache	21	1.3 (0.9, 2)
Fatigue	19	1.2 (0.8, 1.8)
Neurological problem	15	0.9 (0.6, 1.5)
Nausea, vomiting	10	0.6 (0.3, 1.1)
Loose motion/abdominal discomfort	15	0.9 (0.6, 1.5)
Infection	8	0.5 (0.3, 1)
Cancer, stroke liver & kidney problem	8	0.5 (0.3, 1)
Lung problem	7	0.4 (0.2, 0.9)
Death	7	0.4 (0.2, 0.9)
Fever	5	0.3 (0.1, 0.7)
Stroke	2	0.1 (0, 0.5)
Allergy/rash	2	0.1 (0, 0.5)
Gynecological problem	2	0.1 (0, 0.5)
Mineral deficiency	1	0.1 (0, 0.4)
No response	1090	68.1 (65.8, 70.4)

**Table 5:** COVID-19 illness among the general population during the COVID-19 pandemic in Bangladesh.

COVID-19 Illness	n	% (95% CI)
Tested for COVID-19 since the start of COVID-19 pandemic (Self)		
Yes	111	6.0 (5.0, 7.2)
No	1665	89.8 (88.3, 91.1)
Missing/no response	78	4.2 (3.4, 5.2)
COVID-19 test result (n = 111) (self)		
Positive	16	14.4 (9.1, 22.1)
Unknown	2	1.8 (0.5, 6.3)
COVID-19 testing facility type (n = 111) (self)		
Public	90	81.1 (72.8, 87.3)
Private	18	16.2 (10.5, 24.2)
Both	3	2.7 (0.9, 7.6)
Hospitalization required for COVID-19 (n = 111) (self)		
Yes	4	3.6 (1.4, 8.9)
No	107	96.4 (91.1, 98.6)
**COVID-19 information of other family members**		
Number of family members tested for COVID-19 since the start of the COVID-19 pandemic		
1	68	3.7 (2.9, 4.6)
2	25	1.3 (0.9, 2)
3	20	1.1 (0.7, 1.7)
4	11	0.6 (0.3, 1.1)
>5	16	0.9 (0.5, 1.4)
No	1633	88.1 (86.5, 89.5)
Missing/no response	81	4.4 (3.5, 5.4)
Mean Number of family members tested for COVID-19 (Range)	2.3 (1–15)	
The COVID-19 test result of family members (n = 141)		
Negative	111	78.7 (71.3, 84.7)
One to three members positive	18	12.8 (8.2, 19.3)
Missing/no response	12	8.5 (4.9, 14.3)
Hospitalization of family members due to Corona (n = 141)		
Yes	7	5.0 (2.4, 9.9)
No	133	94.3 (89.2, 97.1)
Missing/no response	1	0.7 (0, 3.9)
Death of family members due to COVID-19 (n = 141)		
Yes	5	3.5 (1.5, 8)
No	133	94.3 (89.2, 97.1)
Missing/no response	3	2.1 (0.7, 6.1)

## Data Availability

The data presented in this survey are available on reasonable request from icddr,b’s research administration through the corresponding author. The data are not publicly available due to privacy restrictions and icddr,b policy.

## References

[1] Vallin M., Polyzoi M., Marrone G., Klintz S.R., Wisell K.T., Lundborg C.S. (2016). Knowledge and Attitudes towards Antibiotic Use and Resistance—A Latent Class Analysis of a Swedish Population-Based Sample. PLoS ONE.

[2] Davies J., Davies D. (2010). Origins and Evolution of Antibiotic Resistance. Microbiol. Mol. Biol. Rev..

[3] O’Neill J. (2016). Tackling Drug-Resistant Infections Globally: Final Report and Recommendations.

[4] World Health Organization (2019). Global Action Plan on Antimicrobial Resistance. 2015.

[5] Lu H., Stratton C.W., Tang Y.-W. (2020). Outbreak of pneumonia of unknown etiology in Wuhan, China: The mystery and the miracle. J. Med. Virol..

[6] Huang C., Wang Y., Li X., Ren L., Zhao J., Hu Y., Zhang L., Fan G., Xu J., Gu X. (2020). Clinical features of patients infected with 2019 novel coronavirus in Wuhan, China. Lancet.

[7] Xu Z., Shi L., Wang Y., Zhang J., Huang L., Zhang C., Liu S., Zhao P., Liu H., Zhu L. (2020). Pathological findings of COVID-19 associated with acute respiratory distress syndrome. Lancet Respir. Med..

[8] WHO (2020). WHO Timeline-COVID-19. https://www.who.int/news-room/detail/08-04-2020-who-timeline---covid-19.

[9] Beović B., Doušak M., Ferreira-Coimbra J., Nadrah K., Rubulotta F., Belliato M., Berger-Estilita J., Ayoade F., Rello J., Erdem H. (2020). Antibiotic use in patients with COVID-19: A ‘snapshot’ Infectious Diseases International Research Initiative (ID-IRI) survey. J. Antimicrob. Chemother..

[10] Verroken A., Scohy A., Gerard L., Wittebole X., Collienne C., Laterre P.-F. (2020). Co-infections in COVID-19 critically ill and antibiotic management: A prospective cohort analysis. Crit. Care.

[11] Nestler M.J., Godbout E., Lee K., Kim J., Noda A.J., Taylor P., Pryor R., Markley J.D., Doll M., Bearman G. (2020). Impact of COVID-19 on pneumonia-focused antibiotic use at an academic medical center. Infect. Control. Hosp. Epidemiol..

[12] Clancy C.J., Nguyen M.H. (2020). Coronavirus Disease 2019, Superinfections, and Antimicrobial Development: What Can We Expect?. Clin. Infect. Dis..

[13] Huttner B., Catho G., Pano-Pardo J., Pulcini C., Schouten J. (2020). COVID-19: Don’t neglect antimicrobial stewardship principles!. Clin. Microbiol. Infect..

[14] Gagliotti C., Buttazzi R., Ricchizzi E., Di Mario S., Tedeschi S., Moro M.L. (2021). Community use of antibiotics during the COVID-19 lockdown. Infect. Dis..

[15] Butler C.C., Dorward J., Yu L.-M., Gbinigie O., Hayward G., Saville B.R., Van Hecke O., Berry N., Detry M., Saunders C. (2021). Azithromycin for community treatment of suspected COVID-19 in people at increased risk of an adverse clinical course in the UK (PRINCIPLE): A randomised, controlled, open-label, adaptive platform trial. Lancet.

[16] Chang C., Lee M., Lee J., Lee N., Ng T., Shafie A., Thong K. (2021). Public KAP towards COVID-19 and Antibiotics Resistance: A Malaysian Survey of Knowledge and Awareness. Int. J. Environ. Res. Public Health.

[17] Elsayed A.A., Darwish S.F., Zewail M.B., Mohammed M., Saeed H., Rabea H. (2021). Antibiotic misuse and compliance with infection control measures during COVID-19 pandemic in community pharmacies in Egypt. Int. J. Clin. Pract..

[18] Hicks J.P., Latham S.M., Huque R., Das M., Newell J., Abdullah S.M., Al Azdi Z., Jahan I., Rassi C., Hamade P. (2021). Antibiotic practices among household members and their domestic animals within rural communities in Cumilla district, Bangladesh: A cross-sectional survey. BMC Public Health.

[19] Das P., Martin D., Banu S., Rahman M., Chisti M., Friedman M. (2020). Antibiotic use of patients having acute febrile illness prior to their hospital attendance in Bangladesh. Int. J. Infect. Dis..

[20] Mah-E-Muneer S., Hassan Z., Biswas A.A.J., Rahman F., Akhtar Z., Das P., Islam A., Chowdhury F. (2021). Use of Antimicrobials among Suspected COVID-19 Patients at Selected Hospitals, Bangladesh: Findings from the First Wave of COVID-19 Pandemic. Antibiotics.

[21] Lucas P.J., Uddin M.R., Khisa N., Akter S.M.S., Unicomb L., Nahar P., Islam M.A., Alam Nizame F., Rousham E.K. (2019). Pathways to antibiotics in Bangladesh: A qualitative study investigating how and when households access medicine including antibiotics for humans or animals when they are ill. PLoS ONE.

[22] Liu B., Brotherton J.M., Shellard D., Donovan B., Saville M., Kaldor J.M. (2011). Mobile phones are a viable option for surveying young Australian women: A comparison of two telephone survey methods. BMC Med. Res. Methodol..

[23] Iachan R., Pierannunzi C., Healey K., Greenlund K.J., Town M. (2016). National weighting of data from the Behavioral Risk Factor Surveillance System (BRFSS). BMC Med. Res. Methodol..

[24] Brick J.M., Edwards W.S., Lee S. (2007). Sampling telephone numbers and adults, interview length, and weighting in the California Health Interview Survey cell phone pilot study. Public Opin. Q..

[25] BTRC Teledensity & Internet Penetration at the End of May, 2021. Teledensity (Voice + Internet Subscription) in Bangladesh May, 2021. http://www.btrc.gov.bd/teledensity-internet-penetration.

[26] BTRC Mobile Phone Subscribers in Bangladesh May, 2021. http://www.btrc.gov.bd/content/mobile-phone-subscribers-bangladesh-may-2021.

[27] Harris D., Grimshaw J., Lemon J., Russell I.T., Taylor R. (1993). The Use of a Computer-assisted Telephone Interview Technique in a General Practice Research Study. Fam. Pract..

[28] Gibson D.G., Pereira A., A Farrenkopf B., Labrique A.B., Pariyo G.W., A Hyder A. (2017). Mobile Phone Surveys for Collecting Population-Level Estimates in Low- and Middle-Income Countries: A Literature Review. J. Med. Internet Res..

[29] Choi B.C. (2004). Computer assisted telephone interviewing (CATI) for health surveys in public health surveillance: Methodological issues and challenges ahead. Chronic Dis. Can..

[30] Hassan Z., Monjur M.R., Biswas A.A.J., Chowdhury F., Kafi M.A.H., Braithwaite J., Jaffe A., Homaira N. (2021). Antibiotic use for acute respiratory infections among under-5 children in Bangladesh: A population-based survey. BMJ Glob. Health.

[31] Naing L., Winn T., Rusli B.N. (2006). Practical issues in calculating the sample size for prevalence studies. Arch. Orofac. Sci..

[32] Nasir M., Chowdhury S., Zahan T. (2020). Self-medication during COVID-19 outbreak: A cross sectional online survey in Dhaka city. Int. J. Basic Clin. Pharmacol..

[33] Langford B.J., So M., Raybardhan S., Leung V., Soucy J.-P.R., Westwood D., Daneman N., MacFadden D.R. (2021). Antibiotic prescribing in patients with COVID-19: Rapid review and meta-analysis. Clin. Microbiol. Infect..

[34] Sermo.com Breaking Results: Sermo’s COVID-19 Real Time Barometer Study. https://public-cdn.sermo.com/covid19/c8/be4e/4edbd4/dbd4ba4ac5a3b3d9a479f99cc5/wave-i-sermo-covid-19-global-analysis-final.pdf.

[35] Saha T., Saha T. (2018). Awareness level of patients regarding usage of antibiotics in a slum Area of Dhaka City, Bangladesh. SSRG Int. J. Med. Sci..

[36] Haque M.U., Kumar A., Barik S.A., Islam M.A.U. (2017). Prevalence, practice and irrationality of self-medicated antibiotics among people in northern and southern region of Bangladesh. Europe.

[37] Biswas M., Roy M.N., Manik I.N., Hossain S., Alam Tapu S.M.T., Moniruzzaman M., Sultana S. (2014). Self medicated antibiotics in Bangladesh: A cross-sectional health survey conducted in the Rajshahi City. BMC Public Health.

[38] Alam N., Saffoon N., Uddin R. (2015). Self-medication among medical and pharmacy students in Bangladesh. BMC Res. Notes.

[39] World Health Organization (2020). Clinical Management of COVID-19: Interim Guidance, 27 May 2020.

[40] Abelenda-Alonso G., Padullés A., Rombauts A., Gudiol C., Pujol M., Alvarez-Pouso C., Jodar R., Carratalà J. (2020). Antibiotic prescription during the COVID-19 pandemic: A biphasic pattern. Infect. Control. Hosp. Epidemiol..

[41] World Health Organization (2019). AWaRe Policy Brief.

